# Weston's Metal

**Published:** 1870-04

**Authors:** 


*Weston's Metal.*—We have tested this metal in the case of entire lower sets and, are inclined to the belief that it is superior to anything of the kind which has yet been brought to the notice of the profession. We advise a trial of it by those who find objection to the lighter materials, such as rubber for example, or to the heavier materials such as the pure tin base; as it is claimed for this metal that a plate of it when finished need weigh no more than one of gold.

There is no doubt but that it is stronger, and will keep its color better in the mouth than any of the cast plates which have been in use, except perhaps aluminum, which can only be used in this way combined with an alloy of tin and silver to connect the teeth to the plate.

The following instructions are given for casting Weston's metal:

"Suppose the case to be a full Lower Plate of Gum Teeth,—those intended for Rubber are the only ones adapted to this work. Grind off the thin edges of the Gums( that part intended in Rubber work to be covered by the rim or band) to a right angle with the Labial and Buccal surfaces of the Teeth; the object of this as to prevent the Gum cracking by unequal expansion or contraction. Next upon a cast made of equal parts of Plaster of Paris and clean pulverized Soap Stone (Whiting, Pumice Stone, or clean fine Sand will answer as a substitute for Soap Stone), arrange the teeth in Wax, precisely as you would have them when finished. In grinding the joints' bring the body of the Gum squarely together, as closely as at the edges. Avoid using too much Wax.

When finishing with Wax, be sure to cover the pins well; and in arranging it for the rim, be careful not to let it overlap the Gum. By cutting away all superfluous Wax, and taking pains to make the Wax Plate perfectly smooth, much time and labor can be saved in polishing the piece after it is cast. The Plate now being ready for the Flask, invest the same as for Rubber, in equal parts of Plaster and fine clean Sand. The Flasks used for Rubber will answer, by cutting with a coarse half.round file an opening five eights of an inch in diameter, in the posterior part of the Flask for a gateway. Cut a smaller hole in the opposite side for a vent. When the Plaster in the Flask has set, warm it before opening, so as to soften but not melt the Wax; open the Flask and remove as much of the Wax as possible without marring the Plaster. After doing this in the usual way, any Wax that remains must be carefully washed away by pouring boiling water into the Flask. Next cut a funnel-shaped Gate in the Plaster, through which to pour the Metal; this may be ¼ inch in diameter, where it opens in the Mould, directly posterior to the last Molars; the Vent to be one-half the size of the Gate and directly opposite it. Dry the Flask thoroughly from three to five hours, it must not be used till perfectly dry. Any moisture remaining in it can be easily detected by holding a mirror over the venthole. Now melt the Metal carefully in a clean iron ladle,—do not get it too hot; with the Flask heated so that it can barely he held in the hand, pour the Metal. When quite cooled, open the Flask and remove the Plate. If the piece is as it should be on coming out of the Flask, it will require little or no scraping or filing; use Sand Paper first; next Felt Wheels, and Pumice Stone; and finish with Felt Wheels, and the very finest Brush Wheels and Whiting.

These instructions, carefully followed are applicable to partial Upper and Lower Sets, Plain or Gum Teeth.

To replace a broken block, cut out the broken pieces and fit the block the same as for Rubber work; fill the dovetail with Wax: invest in Plaster and Sand; open the Flask; wash out the Wax; cut a Gate and a Vent; dry carefully; then heat the case to 200° or 210° Fahrenheit. Now pour the Metal in the Gate. If the Gate and the Vent are large enough, and the Case properly dried, the piece when finished will be as good as new.

A plain or single Gum Tooth may be attached when broken off, or added anew, by investing as for Gold or Silver; dry out and heat the piece; moisten the parts to be united with Muriate Zinc; use Blow-Pipe or Soldering Iron. (Solder with the same Metal.) The Metal melts at 440° Fahrenheit."

